# Exploring Determinants of Sustained Participation in New Sports: The Impact of YouTube Engagement and Educator Support

**DOI:** 10.3390/bs14100914

**Published:** 2024-10-08

**Authors:** Dohun Kim, Yunduk Jeong

**Affiliations:** 1 Department of Sports and Welfare, Korea National University of Transportation, Chungju 27469, Republic of Korea; kpga0000@hanmail.net; 2 College of General Education, Kookmin University, Seoul 02707, Republic of Korea

**Keywords:** new sports, theory of planned behavior, social support, media interest, behavioral intentions

## Abstract

This study explored the determinants of sustained participation in new sports by applying the Theory of Planned Behavior (TPB) and examining the moderating role of social support, as well as the influence of media interest. This present study aimed to contribute to the promotion and effective adoption of new sports by providing valuable data for new-sports educators to teach these activities. A survey was conducted among 313 university students enrolled in new-sports classes across five universities in Korea, utilizing a convenience sampling method. Using SPSS 26.0 and AMOS 26.0, we conducted frequency, correlation, and reliability analyses, followed by confirmatory factor analysis (CFA) to validate the measurement model and structural equation modeling (SEM) to examine the relationships between media interest, attitudes, subjective norms, perceived behavioral control (PBC), and behavioral intentions. The software Jamovi version 2.3.21 was employed to analyze the moderating effects of social support from educators on these relationships. The findings reveal that media interest significantly influenced the attitudes, which, in turn, along with subjective norms and PBC, affected the behavioral intentions. Moreover, the social support from educators moderated the effects of attitudes, subjective norms, and PBC on the behavioral intentions. This study concluded that leveraging media interest and enhancing social support can effectively promote engagement and sustained participation in new sports. These insights can offer practical strategies for stakeholders aiming to increase participation rates in new-sports activities.

## 1. Introduction

The popularity of traditional sports, such as soccer, baseball, basketball, golf, and hockey, has been extensively documented, and they hold significant cultural importance worldwide [1]. Despite the widespread appeal of these traditional sports, various new sports have emerged, combining elements of established sports with novel twists to create engaging and inclusive formats [2]. New sports are innovative activities that modify traditional sports to improve the safety, accessibility, and participation for individuals of all ages. By prioritizing enjoyment, these activities create a supportive environment for participants. Unlike traditional sports, new sports emphasize inclusivity and often focus on participation rather than competition [3]. Examples of new sports include tee ball, tchoukball, sports stacking, kin-ball, and floorball, which modify traditional rules to promote inclusivity. The specific types of new sports, along with their rules and instructions, are summarized in Table 1. Such adaptations make these sports more appealing and safer for broader audiences. The pursuit of legitimacy by new-sports organizers through the formation of governing bodies further emphasizes their distinct characteristics compared with traditional sports [2].

As these new sports gain recognition, their potential to address modern health challenges, such as rising obesity rates and declining physical fitness levels among college students, becomes increasingly evident. The rising obesity rates and declining physical fitness levels among college students are alarming. Despite these trends, physical education classes in colleges have predominantly focused on traditional competitive sports aimed at enhancing students’ physical skills [4]. The emphasis on traditional competitive sports, which primarily focuses on physical performance, has proven to be a significant barrier for female and overweight college students. These students often feel uncomfortable or discouraged in highly competitive environments, leading to decreased participation and engagement in physical education classes [5]. Consequently, this decline in participation exacerbates the deterioration of students’ overall health and fitness. In response to these issues, there has been a shift toward implementing student-centered sports models, such as new sports, in college physical education [6].

New sports are designed to partially adapt traditional sports to match students’ abilities, offering a variety of modified sports that cater to a wider range of participants. These sports shift the focus from competition to new values, such as challenge and experience. This change can address many issues inherent in traditional school sports, such as elitism and a win-at-all-costs mentality, providing a more inclusive and practical alternative [7].

The definitions of new sports vary among scholars, with interpretations differing based on the emphasis on specific aspects. Kim [8] categorized the ‘new’ in new sports into four primary meanings: novelty, re-creation, anti-traditional, and alternative. Novelty refers to the introduction of new activities; re-creation involves simplifying traditional sports to create new forms; anti-traditional highlights the differences from modern sports; and alternative signifies a shift toward addressing competitive, male-dominated sports cultures [9,10].

This evolving understanding of new sports has prompted a reassessment of traditional physical education, leading to significant changes in school sports programs [9]. In this context, ‘physical education’ refers to structured programs designed to develop students’ physical fitness, motor skills, and knowledge of healthy lifestyles through active participation in various physical activities. ‘New-sports educators’ are those who adopt and teach emerging sports with the goal of helping students increase their physical activity participation. Consequently, the Ministry of Education has officially recognized new sports in the curriculum, reflecting a growing trend of incorporating these activities to better meet students’ evolving needs and preferences.

Despite the increasing integration of new sports into educational settings, there is a need for academic exploration into the factors influencing students’ intentions to continue participating in these activities. A recent ethnographic study explored the cultural formation process in a new-sports major at a university, highlighting the challenges and growth experienced by students [11]. The study emphasized the educational value of new sports and the students’ ability to shape their own culture through collaboration and perseverance. Previous studies utilized the Theory of Planned Behavior (TPB) to examine the factors affecting behavioral intentions, in particular, attitudes, subjective norms, and perceived behavioral control (PBC) [12,13]. This study aimed to determine whether these three variables, grounded in the TPB, impact university students’ intention to continue participating in new sports.

To address the limitations in previous research, this study also investigated whether interest in YouTube videos related to new sports positively influenced attitudes toward these activities. While engaging and informative online content can enhance viewers’ understanding and appreciation of new sports, it is crucial to ensure that students access credible and safe sources. To support this, educators should recommend reliable YouTube channels and videos that are recognized for their accuracy and safety, aiming to guide students toward positive content and minimize exposure to misinformation or unsafe sports representations.

This study analyzed whether social support from new-sports educators, including both professors and instructors who teach new sports at universities, moderates the effects of attitudes, subjective norms, and PBC on behavioral intentions to engage in new sports. Social support is crucial because encouragement and guidance from educators can significantly impact students’ motivation and confidence when participating in new sports [14]. Positive reinforcement and a supportive environment can help overcome barriers to participation, making students more likely to develop a sustained interest in these activities.

With these points in mind, this study aimed to explore the determinants of sustained participation in new sports, leveraging the TPB and examining the moderating role of social support and the influence of media interest. Ultimately, this study aimed to contribute to the revitalization of new sports and provide foundational data for new-sports YouTubers and educators to aid them in their efforts to promote and teach new sports effectively. By understanding these determinants, stakeholders can develop strategies to enhance the engagement and sustained participation in new-sports activities.

## 2. Theoretical Background and Research Hypotheses

### 2.1. The Theory of Reasoned Action (TRA) and Theory of Planned Behavior (TPB)

The Theory of Reasoned Action (TRA) and the Theory of Planned Behavior (TPB) are pivotal social and psychological models that explain human behavior across various environments [15]. The TRA, developed before the TPB, primarily examines the factors influencing behaviors and intentions [16]. In particular, it suggests that an individual’s behavior stems from their intention to perform that behavior, with these intentions serving as predictors of actual behavior. According to the TRA, behavior is influenced by the intention to act, which is determined by two key components: attitudes and subjective norms [17]. This model underscores that the intentions that drive a person’s actions are pivotal in predicting their actual behavior. For instance, if someone intends to participate in a new sport, this intention, shaped by their attitude toward the activity and the subjective norms surrounding it, will likely result in actual participation in the sport.

According to the TRA framework, attitudes are derived from the belief that a specific action will lead to particular outcomes and can determine an individual’s feelings about these outcomes [18]. Essentially, attitudes refer to an individual’s positive or negative evaluation of performing a behavior [19]. In the context of new sports, an individual’s attitude toward participating in such activities is crucial. For example, a person might have a favorable attitude toward engaging in a new sport if they believe it will be enjoyable and beneficial to their health. Positive attitudes can significantly enhance the likelihood of sustained participation in new sports, as individuals are more inclined to engage in activities they perceive positively.

Subjective norms in the TRA framework are related to the perceived social pressure to engage or not engage in a behavior [20]. When an individual considers participating in new sports, the perspectives and support of significant people in their lives—such as family, friends, and colleagues—play a critical role. If these influential people support the individual’s participation, the individual will be more likely to engage in the activity. Conversely, a lack of support can deter participation. Subjective norms, thus, represent perceived social expectations and the individual’s motivation to conform to these expectations [12].

However, while the TRA effectively explains behaviors that are under complete volitional control, it has its limitations in situations where control is less certain or influenced by external factors [21]. Recognizing these limitations, Ajzen [17] extended the TRA to develop the TPB, introducing the construct of PBC. PBC reflects an individual’s perception of their ability to perform a given behavior, considering both internal factors (such as skills and confidence) and external factors (such as resources and opportunities) [15]. The TPB, thus, provides a more comprehensive framework by integrating these additional elements, which are crucial for understanding behaviors that are not entirely within the individual’s control.

In the context of new sports, PBC plays a significant role in determining participation. It includes an individual’s confidence in their ability to participate despite potential barriers, such as a lack of facilities, time constraints, or limited resources. Factors influencing PBC can vary widely, from personal confidence and perceived skills to external conditions, like the availability of equipment and social support. For example, an individual might feel confident in their ability to play a new sport if they can access the necessary equipment and feel supported by friends and family. On the other hand, a lack of these resources can negatively impact their PBC and, consequently, their participation intentions.

### 2.2. Interest

Interest is fundamentally defined as a positive feeling toward an activity or object, or the inclination to pursue and engage in activities perceived as valuable [22]. It represents an individual’s behavioral tendency to show curiosity and dedication toward specific tasks or experiences. Notably, an initial interest in an activity or subject is not always necessary; interest can develop and evolve during the engagement process [23]. For instance, an individual might not be initially interested in a new sport, but through participation and exposure, they might develop a keen interest in it over time. This dynamic nature of interest makes it a critical concept in understanding how individuals choose and sustain engagement in various activities.

Interest plays a significant role in the cognitive processes involved in selecting, processing, and prioritizing information [22]. It helps determine where individuals allocate their attention and cognitive resources, making it a crucial factor in various cognitive tasks. For example, when a student is interested in a subject, they are more likely to pay attention, process information more deeply, and retain that information longer. This heightened state of attention and cognitive engagement can lead to better learning outcomes and sustained participation in the activity. Thus, understanding interest is essential for developing strategies to enhance cognitive performance and sustained engagement in educational and recreational activities.

From a motivational perspective, interest is described as a state of ongoing or recurring engagement with a particular subject or activity [24]. This engagement is not just a fleeting moment of curiosity but rather a sustained involvement that drives individuals to seek further interaction and mastery [25]. For example, in the context of new sports, if a student finds a sport intriguing and enjoyable, they are more likely to continue participating and eventually excel in it. Situational factors, such as the presence of a supportive environment and positive reinforcement, can significantly influence the development and maintenance of interest. Media interest, particularly through YouTube videos, plays a critical role in this context, as exposure through various media channels can spark and sustain interest in new sports.

Interest is not just a standalone emotion but rather interacts dynamically with other emotional and cognitive processes [26]. It is closely related to curiosity, which drives individuals to explore new and novel experiences [27]. While curiosity seeks novelty, interest often builds on this by developing a deeper appreciation and valuation of the activity or subject [28]. For instance, a person might initially be curious about a new sport they saw on YouTube. As they learn more about it and start participating, this curiosity can transform into a sustained interest, driven by positive experiences and reinforcement. This process highlights the importance of media in fostering the initial curiosity and converting it into long-term interest. The role of YouTube and other social media platforms is especially significant in this regard, as they can provide accessible and engaging content that introduces new sports to a wider audience, thereby cultivating interest and participation. Research showed that social network sports communities positively impact individuals’ sports attitudes and loyalty, suggesting that social media plays a crucial role in shaping sports engagement [29].

The development of interest involves a complex interplay between individual predispositions and environmental factors. It often starts with an initial trigger—something that captures attention and sparks curiosity [28]. Then, through continued engagement and positive experiences, this initial situational interest can develop into a more stable and enduring personal interest [24]. For example, a student might be introduced to a new sport through a school program or media exposure. Positive reinforcement from peers, teachers, and family, along with personal achievements in the sport, can help sustain and deepen this interest, leading to ongoing participation and involvement. Additionally, YouTube videos related to sports can serve as a continual source of inspiration and information, reinforcing interest and engagement through visually appealing and informative content, as seen in studies that showed the positive influence of social network communities on sports attitudes [29].

In educational psychology, interest is viewed as a motivational construct that emerges from the interaction between the individual and the environment [24]. It is often categorized into two types: situational interest, which is temporary and context dependent, and personal interest, which is more enduring and individual specific [25]. Situational interest can be sparked by external factors, such as a compelling presentation or a novel activity, while personal interest is built over time through repeated positive experiences and reinforcement [24,28]. In the context of new sports, creating engaging and supportive environments can help convert situational interest into sustained personal interest, fostering long-term engagement and participation. The influence of media, particularly platforms like YouTube, can play a significant role in this process by continuously exposing individuals to new and exciting content related to sports, which can help foster positive attitudes and long-term loyalty toward sports participation [29].

### 2.3. Social Support

Social support is described as the perceived or tangible assistance individuals receive from their social networks, which can include both formal support systems and informal personal connections [30]. This support is crucial in enhancing individuals’ adaptive capacities and overall well-being [31]. Social support is typically categorized into instrumental and emotional dimensions.

Instrumental support involves the provision of practical assistance with everyday tasks or during times of need [32]. In the context of new sports, instrumental support might include providing the necessary equipment, arranging transportation to and from practice sessions or competitions, and assisting with time management to balance sports with other responsibilities. This type of support extends beyond financial assistance to include tangible resources and services that help individuals to engage in sports activities and pursue their interests. For example, a new-sports instructor might ensure that participants have access to training facilities, coordinate travel for events, and offer personalized training schedules. Such practical support can significantly reduce barriers to participation, enabling individuals to focus on their performance and development in new sports.

Emotional support encompasses the sharing of emotions, empathy, and affirmation within personal relationships [33]. It includes social interactions where individuals discuss personal issues or feelings and receive advice and reassurance [34]. For participants in new sports, emotional support from instructors or peers can involve creating an encouraging and empathetic environment where they feel comfortable sharing their experiences and challenges. Instructors can demonstrate empathy by understanding and acknowledging the participants’ struggles and successes, thereby validating their efforts and contributions to the sport. This emotional rapport helps build a sense of community and belonging among participants, fostering a supportive atmosphere that can motivate them to persist in their sports involvement despite obstacles.

In the realm of new sports, the role of social support is particularly crucial. Instructors and mentors provide not only the necessary resources and practical assistance but also the emotional encouragement needed to sustain long-term engagement. For instance, a new-sports coach might regularly check in with participants to discuss their progress and any difficulties they are facing, offer motivational talks, and celebrate their achievements. This dual approach of instrumental and emotional support can, in turn, greatly enhance the participants’ experience and commitment to the sport.

### 2.4. Research Hypotheses Development

Several studies demonstrated the positive impact of interest on attitudes across different contexts. For instance, Kim [35] found that using movies for shadowing activities in English learning increased students’ interest and led to a more positive attitude toward English classes, which were previously perceived as boring and rigid. This suggests that heightened interest can shift students’ attitudes in a favorable direction. Choi and Lee [36] identified that college students’ interest in general dance education significantly influenced their learning attitudes, with higher interest correlating with more positive learning behaviors. This finding supports the notion that interest is a critical emotional factor that enhances engagement and shapes favorable attitudes toward learning activities. In a similar vein, Choi [37] determined that consumers who exhibited higher interest in sales promotions developed more positive attitudes toward accepting promotions, which, in turn, positively influenced their satisfaction with and loyalty to shopping malls. Choi’s study further indicates that interest plays a crucial role in forming positive attitudes in various behavioral contexts. Drawing on these findings, we hypothesized the following:

**Hypothesis** **H1.** *Interest positively influences attitudes.*

Previous research has consistently reported that attitudes, subjective norms, and PBC significantly influence behavioral intentions, as supported by the TPB. For example, Wang et al. [38] explored the psychosocial determinants of physical activity intentions among patients with coronary heart disease and found that both attitudes and PBC positively influenced the intention to engage in physical activity, while subjective norms did not show a significant effect. Seonwoo and Jeong [15] investigated the factors affecting career pursuit intentions among Taekwondo athletes and revealed that attitudes, subjective norms, and PBC all positively impacted the athletes’ intentions to continue pursuing a career in Taekwondo, indicating the broad applicability of the TPB in sports contexts. Yu and Jeong [13] focused on aspiring esports athletes, examining how attitudes, subjective norms, and PBC influenced their career pursuit intentions. Their results confirmed that all three components were significant predictors of career intentions, with subjective norms being the most influential. Schuster et al. [39] studied caregivers’ intentions to encourage their children to walk to school and reported that subjective norms and PBC significantly influenced behavioral intentions, while attitudes had a weaker yet still positive effect, reinforcing the importance of these TPB components in motivating health-related behaviors. Based on these findings, the following research hypotheses were proposed:

**Hypothesis** **H2-1.** *Attitude positively influences behavioral intentions.*

**Hypothesis** **H2-2.** *Subjective norms positively influence behavioral intentions.*

**Hypothesis** **H2-3.** *Perceived behavioral control positively influences behavioral intentions.*

In addition to attitudes, subjective norms, and PBC, social support has been recognized as a critical element that can enhance an individual’s intention to engage in certain behaviors. Jeong and Kim [40] explored the role of social support in fostering the intention to continue exercising, demonstrating that various forms of social support—emotional, informational, and instrumental—can significantly strengthen individuals’ intentions. However, the interaction between social support and the traditional predictors of behavioral intention—such as attitudes, subjective norms, and PBC—has not been thoroughly examined. It is plausible that social support can serve as a moderating factor, enhancing the positive effects of attitudes, subjective norms, and PBC on behavioral intentions. For instance, when individuals perceive strong social support, the influence of their positive attitudes, the perceived social pressures (subjective norms), and their confidence in performing the behavior (PBC) on their intentions might be amplified. Conversely, in the absence of adequate social support, these influences might be weaker.

Therefore, this study proposed the following hypotheses:

**Hypothesis** **H3-1.** *The effect of attitudes on behavioral intentions is moderated by social support, with a stronger relationship when social support is high.*

**Hypothesis** **H3-2.** *The effect of subjective norms on behavioral intentions is moderated by social support, with a stronger relationship when social support is high.*

**Hypothesis** **H3-3.** *The effect of perceived behavioral control on behavioral intentions is moderated by social support, with a stronger relationship when social support is high.*

**Figure 1:**
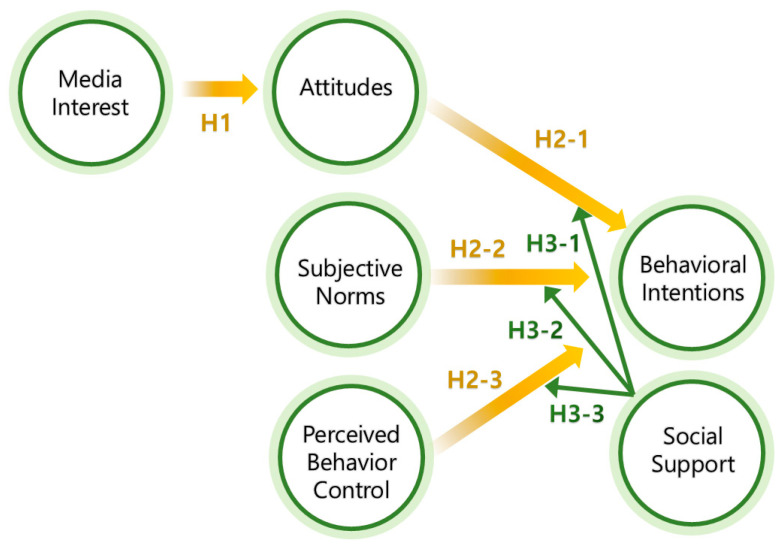
Proposed conceptual model.

After reviewing the available literature, the current study utilized the conceptual framework depicted in Figure 1.

## 3. Method

### 3.1. Sampling and Data Collection

This study employed a convenience sampling method due to time and resource constraints, allowing for accessible and practical data collection from university students enrolled in new-sports classes. The researchers first contacted the deans of the selected universities to explain this study’s purpose and objectives. Upon receiving approval, the researchers visited the new-sports classes, which were either part of elective or major courses, and conducted face-to-face surveys with the students. Only students who consented to participate were included, and the research’s purpose and objectives were thoroughly explained to them before completing the questionnaire. To ensure confidentiality, the surveys were conducted in private settings, separate from other students. Participants were informed that their responses would remain anonymous and that their participation was voluntary, without any impact on their academic standing or relationships with the instructors. These steps were taken to minimize any potential bias and ensure honest responses.

The demographic analysis of the respondents revealed that 40.6% were male and 59.4% were female. In terms of the academic year, 23.3% of the participants were in their first year, 26.8% were in their second year, 26.2% were in their third year, and 23.6% were in their fourth year. Regarding their experience with new-sports courses, 56.5% of the students had taken one course, 20.4% had taken two, 15.0% had taken three, and 8.0% had taken four courses. Additionally, the frequency of exercise per week varied, with 26.8% of students reporting no exercise, 23.0% exercising once a week, 18.2% twice a week, 15.3% three times a week, and 16.6% exercising four or more times a week.

### 3.2. Measures

The survey included 20 questions related to attitudes, subjective norms, PBC, interest in new sports, social support from educators, and intentions to continue participating in new sports. All measurement scales used in this study were adapted from established academic literature and was validated in previous research. The survey was administered in person using printed questionnaires, and the responses were recorded on a 5-point Likert scale for most variables, with options ranging from ‘strongly disagree’ to ‘strongly agree’. To ensure the students accessed credible and safe content related to new sports, the educators recommended reliable YouTube channels and videos recognized for their accuracy and safety, minimizing exposure to misinformation or unsafe sports representations.

Attitudes toward new sports were measured with three items adapted from Kim et al. [41] and Seonwoo and Jeong [15] using scales ranging from ‘extremely unpleasant’ to ‘extremely pleasant’, ‘extremely unattractive’ to ‘extremely attractive’, and ‘extremely worthless’ to ‘extremely valuable’. Although these scales were validated in previous research, a full psychometric validation process was not conducted in this study due to time constraints. However, reliability tests (Cronbach’s alpha) were performed to ensure internal consistency. Subjective norms were assessed using three items from Yu and Jeong [13], and PBC was measured with three items adapted from Ajzen [17] and Perugini and Bagozzi [42], as these scales have been extensively used in behavioral research and align with the TPB framework. Media interest was measured using items adapted from Chen, Darst, and Pangrazi [43], which are widely used in educational and recreational research to capture students’ engagement and curiosity. Lastly, the scale for social support from educators was adapted from Park [44], as it reflects established social psychology research and was relevant for understanding the role of support in new-sports participation. All items were measured using a 5-point Likert scale. The attitude variable was measured using bipolar scales, while the other variables were measured using scales that ranged from ‘strongly disagree’ to ‘strongly agree’. Although the scales were adapted from established research, further psychometric validation could strengthen the rigor of future research. The detailed survey items and format are provided in Table 2 for full replication.

### 3.3. Data Analysis

The survey data were analyzed using the SPSS 26.0 and AMOS 26.0 software packages. SPSS was utilized for the frequency, correlation, and reliability analyses, while AMOS was used for the confirmatory factor analysis (CFA) and structural equation modeling (SEM). SEM was selected due to its ability to assess the complex relationships between observed and latent variables simultaneously. Additionally, Jamovi was employed to analyze the moderating effects within the data. Rather than pre-selecting specific YouTube videos, educators recommended reliable channels and videos related to new sports and occasionally showed them during class to ensure that participants had access to credible and safe content.

### 3.4. Validity and Reliability

In the present investigation, the CFA with maximum likelihood estimation was performed in AMOS to validate the dimensional structure of the measurement model. The goodness-of-fit indices for the CFA (χ^2^/*df* = 2.985, normed fit index [NFI] = 0.915, incremental fit index [IFI] = 0.923, comparative fit index [CFI] = 0.924, root-mean-square error of approximation [RMSEA] = 0.059) all fell within the suggested thresholds, as outlined by Hooper et al. [45].

To establish the convergent validity, this study computed the factor loadings, construct reliability (CR), and average variance extracted (AVE) using the measurement model. As depicted in Table 1, all factor loadings (which ranged from 0.702 to 0.933) were statistically significant (*p* < 0.001) and exceeded the threshold of 0.50. The CR values (which ranged from 0.854 to 0.961) surpassed the minimum requirement of 0.7, and all AVE values (which ranged from 0.653 to 0.892) exceeded the suggested threshold of 0.5 [46]. Since all CR and AVE values met the specified thresholds, the convergent validity was established.

For the satisfactory discriminant validity, the diagonal elements needed to be greater than the off-diagonal elements, a condition that was met. The comparison of all correlation coefficients with the square roots of the AVE demonstrated satisfactory discriminant validity.

## 4. Results

### 4.1. Model Fit and Structural Model

**Figure 2:**
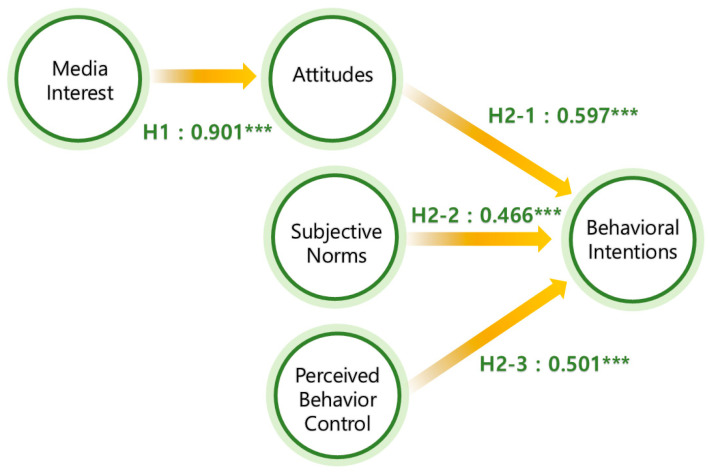
Structural model results. *** *p* < 0.001.

This study employed structural equation modeling (SEM) to examine the proposed relationships between the variables. The model fit indices indicate a good fit (χ^2^/*df* = 2.715, NFI = 0.924, IFI = 0.931, TLI = 0.918, CFI = 0.927, RMSEA = 0.070). Accordingly, this model was used to test the hypotheses. As illustrated in Figure 2, our analysis confirmed a statistically significant link between the media interest and attitudes, with a coefficient of 0.901 (*p* < 0.001), supporting H1. Additionally, H2-1 was validated, as the results reveal a positive correlation between the attitudes and behavioral intentions, with a coefficient of 0.597 (*p* < 0.001). Furthermore, subjective norms were positively associated with the behavioral intentions, confirming H2-2 with a coefficient of 0.466 (*p* < 0.001). Finally, H2-3 was supported, showing a significant correlation between the PBC and behavioral intentions, with a coefficient of 0.501 (*p* < 0.001).

### 4.2. Tests of Moderating Effect

As hypothesized in H3-1, attitudes (Z = 12.33, *p* < 0.001) and social support (Z = 10.96, *p* < 0.001) significantly impacted the behavioral intentions. Furthermore, their interaction (Z = 3.52, *p* < 0.001) also had a significant effect on the behavioral intentions, thus validating H3-1. The independent variable, the moderator, and their interaction all exhibited positive effects, indicating an enhancing influence. Consequently, a simple slope analysis was conducted to explore the variations in the slope between the independent and dependent variables across the three levels of the moderator: low (mean −1 SD), average, and high social support (mean +1 SD). Ultimately, the analysis indicated that social support significantly influenced the behavioral intentions across all three groups (low, average, and high social support) (respectively, Z = 4.92, *p* < 0.001; Z = 12.10, *p* < 0.001; Z = 18.47, *p* < 0.001).

**Figure 3:**
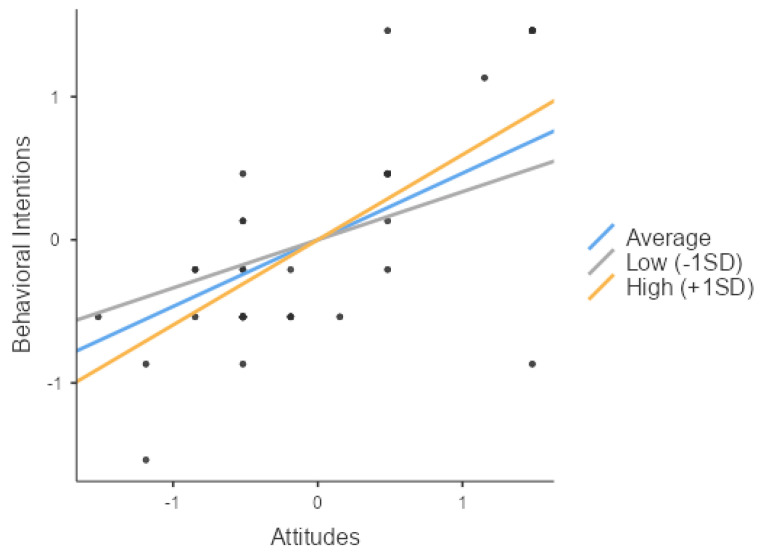
Simple slope plot of the moderating effect of social support on the relationship between attitudes and the behavioral intentions (H3-1).

As Figure 3 illustrates, the slope progressively increases from the low- to the high-support groups, demonstrating that the moderating variable (social support) enhanced the influence of the independent variable (attitudes) on the behavioral intentions.

Similarly, as hypothesized in H3-2, subjective norms (Z = 15.98, *p* < 0.001) and social support (Z = 9.57, *p* < 0.001) significantly impacted the behavioral intentions. Furthermore, their interaction (Z = 6.14, *p* < 0.001) also had a significant effect on the behavioral intentions, thus validating H3-2. The independent variable, the moderator, and their interaction all exhibited positive effects, indicating an enhancing influence. Consequently, a simple slope analysis was conducted to explore the variations in the slope between the independent and dependent variables across three levels of the moderator: low (mean −1 SD), average, and high social support (mean +1 SD). The analysis indicated that social support significantly influenced the behavioral intentions across all three groups (low, average, and high social support) (respectively, Z = 15.32, *p* < 0.001; Z = 7.13, *p* < 0.001; Z = 17.61, *p* < 0.001).

**Figure 4:**
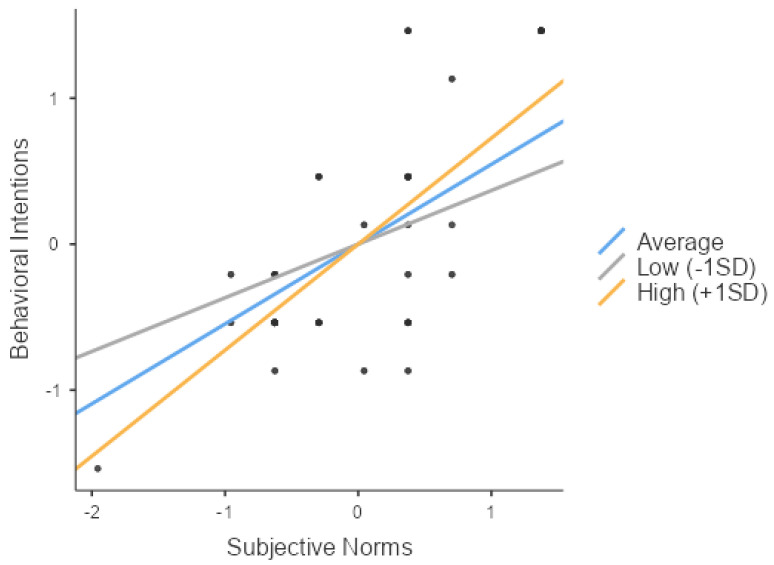
Slope plot of the moderating effect of social support on the relationship between subjective norms and the behavioral intentions (H3-2).

As Figure 4 depicts, the slope progressively increases from the low- to the high-support groups, indicating that the moderating variable (social support) enhanced the influence of the independent variable (subjective norms) on the behavioral intentions.

Next, as hypothesized in H3-3, PBC (Z = 19.59, *p* < 0.001) and social support (Z = 7.00, *p* < 0.001) significantly impacted the behavioral intentions. In addition, their interaction (Z = 10.39, *p* < 0.001) had a significant effect on the behavioral intentions, thus validating H3-3, and the independent variable, the moderator, and their interaction all exhibited positive effects, indicating an enhancing influence. A simple slope analysis was performed to explore the variations in the slope between the independent and dependent variables across the three levels of the moderator, i.e., low, average, and high social support. The analysis confirmed that social support significantly influenced the behavioral intentions across all three groups (low, average, and high social support) (respectively, Z = 18.11, *p* < 0.001; Z = 9.20, *p* < 0.001; Z = 20.60, *p* < 0.001).

**Figure 5:**
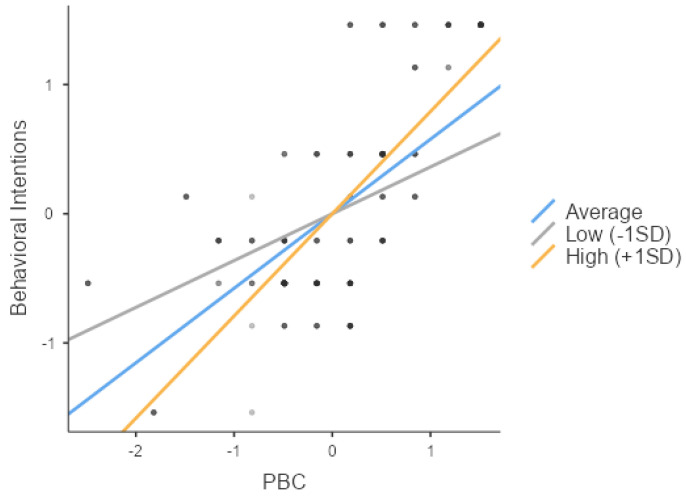
Slope plot of the moderating effect of social support on the relationship between PBC and the behavioral intentions (H3-3).

As shown in Figure 5, the slope progressively increases from the low- to high-support groups, demonstrating that the moderating variable (social support) enhanced the influence of the independent variable (PBC) on the behavioral intentions.

## 5. Discussion and Conclusions

### 5.1. Theoretical Implications

This study confirmed that media interest significantly influenced the attitudes toward new sports, as hypothesized in H1. In other words, when the individuals engaged with media content, such as YouTube videos related to new sports, they were more likely to develop favorable attitudes toward new sports. Our findings are supported by previous studies [35,36,37] similarly suggesting that interest, particularly when stimulated by engaging media, can significantly shape attitudes. This consistency with prior studies reinforces the robustness of our results and highlights the importance of media-driven interest in shaping perceptions in various contexts, including new sports. In particular, the specific impact of media interest on attitudes toward new sports highlights the power of accessible and appealing media content in shaping perceptions. Visual and interactive elements of online videos make the content more relatable and engaging, thereby enhancing viewers’ attitudes toward the new sports featured.

Furthermore, with respect to interest, this study contributes to the existing literature by addressing a gap in the research on the role of media-driven interest in new sports. While previous studies in education and marketing explored interest, how media interest affects attitudes remains underexplored in the context of new sports. This research provides empirical evidence that media interest is a crucial factor in shaping attitudes toward new sports. Finally, the findings suggest several directions for future research on the concept of media interest. For example, researchers can explore how different types of media content affect attitudes and behaviors related to new sports and investigate whether media-generated interest can lead to long-term engagement in new sports. This study lays the groundwork for the further exploration of media interest as a key influence on attitudes toward new sports.

Regarding attitudes and behavioral intentions toward new sports, as hypothesized, this study confirmed a significant relationship. More specifically, the individuals with positive attitudes toward new sports were more likely to develop strong intentions to engage in these activities. This finding aligns with the TPB, which suggests that favorable attitudes are key predictors of the intention to perform a behavior. In this study’s context, the impact of attitudes on behavioral intentions can be attributed to the perceived enjoyment and benefits of new sports. When individuals find these activities exciting and valuable, their positive attitudes enhance their motivation to participate in them. This is consistent with the intrinsic motivation outlined in Self-Determination Theory, where individuals are more likely to engage in activities that fulfill their innate psychological needs for competence and enjoyment, leading to stronger behavioral intentions.

This study adds to the existing literature on attitudes and behavioral intentions by highlighting their relatively unexplored relationship in the context of new sports. Our findings are consistent with previous research in other fields [47,48] that showed that attitudes significantly predicted behavioral intentions. However, the role of attitudes in influencing participation in new sports has not been extensively studied. By demonstrating this link, our study contributes to a better understanding of how attitudes drive engagement in emerging sports activities. Finally, these findings suggest practical implications for promoting new sports. By fostering positive attitudes through targeted campaigns and media exposure, it may be possible to increase participation rates. Future research should explore how different factors, such as social influence and media, interact with attitudes to shape behavioral intentions toward new sports.

Regarding subjective norms, as hypothesized, this study confirmed a significant influence on the behavioral intentions toward new sports. In the context of new sports, the individuals who believed that important people in their lives (such as friends, family, and peers) expected them to engage in new sports were more likely to form strong behavioral intentions to participate in them. This influence of subjective norms on the behavioral intentions could be attributed to the desire to meet social expectations and gain social approval. When the individuals perceived that others around them viewed participation in new sports positively, they were more motivated to align their behavior with these expectations. This aligns with social influence theories, such as Social Cognitive Theory, which suggests that the behavior of individuals is shaped by the expectations and behaviors of those around them. In the context of new sports, this social influence is particularly powerful when the activity is novel or unfamiliar, as individuals often rely on the opinions and expectations of their social circles to guide their participation. Additionally, the Theory of Planned Behavior emphasizes that subjective norms play a key role in shaping intentions, further reinforcing the importance of social support in encouraging participation in new sports.

Our findings regarding subjective norms align with previous research that has consistently demonstrated their importance in shaping behavioral intentions across various domains [49,50], including health behaviors, environmental actions, and consumer choices. For instance, studies in music education [49] highlighted how subjective norms significantly influence students’ intentions to continue participating in musical activities, demonstrating that when individuals perceive that important people in their lives support a particular behavior, they are more likely to develop strong behavioral intentions. Similarly, in environmental behavior research, subjective norms were shown to play a crucial role in fostering pro-environmental intentions by reinforcing social approval and expectations [50]. However, in the context of sports, particularly new sports, the specific role of subjective norms in influencing participation has been less explored. This study contributes to filling this gap by demonstrating that when the individuals believed their social circles expected them to engage in new sports, they were more motivated to participate in these activities, aligning their behavior with these social expectations. By demonstrating the significance of subjective norms in this context, our study contributes to a deeper understanding of the social factors that drive engagement in emerging sports activities. Finally, future research should explore how different social groups and networks influence subjective norms and how these norms interact with other factors, such as attitudes and PBC, to shape behavioral intentions toward new sports.

Regarding PBC and behavioral intentions toward new sports, this study confirmed a significant relationship, as hypothesized. In the context of new sports, individuals who feel they have the necessary skills, time, and access to facilities are more likely to form strong intentions to engage in these activities. PBC’s influence on behavioral intentions can be attributed to the fact that individuals are more inclined to intend to perform behaviors they believe they can successfully execute. This perception of control reduces the psychological barriers to participation and enhances motivation, which aligns with previous studies in various domains, where higher perceived control strengthens one’s confidence to act on their intentions. As proposed by Bandura’s theory of self-efficacy, this sense of control plays a vital role in increasing the likelihood of engaging in new-sports activities.

Our findings align with previous research that consistently showed PBC to be a critical determinant of behavioral intentions across various domains [39,51], including health behaviors, academic performance, and consumer behavior. For example, research on walk-to-school behavior emphasized how PBC influences caregivers’ decisions regarding their child’s physical activity, as it reduces psychological barriers by fostering a sense of control over the behavior [39]. Similarly, studies in halal tourism have highlighted the moderating role of PBC in shaping behavioral intentions, demonstrating that individuals are more likely to act on their intentions when they feel capable of controlling the outcome [51]. However, the specific influence of PBC on participation in new sports has not been as extensively studied. By demonstrating its significant impact in this context, our study contributes to a deeper understanding of the psychological mechanisms that drive engagement in emerging sports activities. Finally, this study’s findings on PBC can provide practical implications for promoting new sports. In particular, to increase participation rates, sports organizations and educators should focus on enhancing individuals’ perceived control over their ability to participate in new sports. With this in mind, future re-search should explore how different elements of PBC, such as self-efficacy and resource availability, interact to influence behavioral intentions toward new sports.

Regarding the variable of social support, this study demonstrated that social support from new-sports educators significantly moderates the relationships between attitudes, subjective norms, PBC, and behavioral intentions toward new sports. Specifically, strong educator support amplifies the impact of these key factors on individuals’ intentions to engage in new sports, highlighting the critical role of educator influence and support in shaping behavioral outcomes.

First, the moderating effect of educator support on the relationship between attitudes and the behavioral intentions suggests that when individuals receive encouragement and guidance from educators, their positive attitudes toward new sports are more likely to translate into strong behavioral intentions. Educator support not only reinforces positive attitudes but also enhances the likelihood that these attitudes will lead to actual participation in new sports. The supportive environment created by educators can help individuals feel more confident and motivated [52], thereby strengthening the connection between their attitudes and intentions.

Second, educator support also moderated the influence of subjective norms on the behavioral intentions. While subjective norms are shaped by the expectations of important people in an individual’s life [53], such as family and friends, the presence of strong educator support can further reinforce these norms. When individuals perceive that their educators, as well as family and friends, expect and support their engagement in new sports, they are more likely to develop strong intentions to participate in these sports. Overall, this finding underscores the importance of educator support in translating perceived social pressures into actual behavioral intentions.

Lastly, the moderating effect of educator support on the relationship between PBC and the behavioral intentions indicates that when individuals feel supported by their educators, their perceived control over engaging in new sports is more likely to lead to strong behavioral intentions. This suggests that educator support can enhance individuals’ confidence in their ability to participate in new sports, thereby making their perceived control more effective in driving behavioral intentions.

### 5.2. Practical Implications

This study can offer valuable practical guidance for enhancing the promotion of new sports by capitalizing on media interest, particularly through platforms like YouTube, and influencing attitudes, subjective norms, PBC, and social support.

First, the notable impact of media interest on attitudes toward new sports highlights the need to effectively use platforms such as YouTube to spark interest and foster positive perceptions. For example, new-sports organizations should prioritize the creation of compelling and informative YouTube content that showcases the unique features and advantages of new sports. This approach can help to cultivate favorable attitudes among viewers and boost their likelihood of engaging in these activities [54]. Such content could include tutorials, success stories, and highlights of the most thrilling aspects of new sports, making them more accessible and appealing to a broader audience.

Additionally, the findings indicate that attitudes toward new sports play a crucial role in shaping behavioral intentions. Consequently, efforts to encourage new sports should prioritize positively influencing potential participants’ attitudes. This can be achieved through targeted efforts, such as endorsements by popular athletes or influencers and community events that offer individuals the chance to experience new sports directly. By fostering positive attitudes, new-sports organizations can increase the probability that individuals will develop strong intentions to participate in new sports.

Moreover, the influence of subjective norms on the behavioral intentions suggests that social influence is a significant factor in the promotion of new sports. Therefore, educators, coaches, and key community figures should actively promote participation in new sports, highlighting the social approval and support associated with such activities. For example, campaigns featuring testimonials from peers and role models who actively participate in new sports can help to create a supportive social environment that encourages others to get involved.

Regarding PBC, its confirmed effect on the behavioral intentions indicates that potential participants must feel confident in their ability to engage in new sports to develop a strong intention to participate in them. To this end, providing the necessary resources, such as accessible facilities, beginner-friendly programs, and clear instructional materials, can help reduce perceived barriers and boost individuals’ perceived control over their participation. New-sports organizations should strive to present new sports as both accessible and achievable, even for those with little or no prior experience.

Lastly, this study found that social support, particularly from educators, plays a critical role in moderating the influence of attitudes, subjective norms, and PBC on behavioral intentions. Crucially, educators who provide encouragement, guidance, and resources can significantly influence individuals’ confidence and motivation to engage in new sports. By offering strong social support, educators help participants to overcome barriers, increase their perceived control, and reinforce their intentions to participate. New-sports organizations should, therefore, focus on training and empowering educators to effectively support and inspire individuals in adopting new sports.

### 5.3. Limitations and Future Research

Despite this study’s contributions, several limitations warrant mentioning for future research. First, this study primarily examined a specific demographic group—university students—which may limit the generalizability of the findings to broader populations. Future research should, therefore, consider exploring diverse age groups and different cultural contexts to better understand the impact of media interest, attitudes, subjective norms, and PBC on new-sports participation across various populations. Second, this research utilized self-reported data to assess attitudes, subjective norms, PBC, and behavioral intentions, which may have been subject to social desirability bias or inaccurate self-assessment. Future studies could incorporate longitudinal designs or objective measures of behavior to validate the findings and reduce the potential biases associated with self-reported data. Third, while this study considered the role of social support from educators, it did not extensively explore the influence of other potential moderators, such as peer influence or personal experience with new sports. Therefore, future research could investigate these additional moderators to provide a more comprehensive understanding of the factors that drive new-sports participation.

## Figures and Tables

**Table 1:** Overview of new sports and their key rules/instructions.

Sport	Brief Rules/Instructions
Tee ball	The ball is placed on a stationary tee and players hit it without a pitcher, making it suitable for beginners.
Tchoukball	Players score by rebounding the ball off a frame and landing it in the opponent’s area. Physical contact is not allowed.
Sports stacking	Players quickly stack and unstack special cups in predetermined sequences, aiming for speed and accuracy.
Kin-ball	Teams work together to keep a large inflatable ball in the air, preventing it from touching the ground, while coordinating strategic plays.
Floorball	Played indoors with a ball and lightweight sticks, this sport emphasizes speed and finesse, with minimal physical contact.
Pickleball	Played with paddles and a perforated plastic ball on a small court, combining elements of tennis, badminton, and table tennis.
Spikeball	Teams hit a small ball onto a net in the center, trying to make it difficult for the opposing team to return it.
Speedminton	Similar to badminton but played without a net and using a heavier shuttlecock to minimize the effects of wind.
Netball	Players pass the ball to teammates in designated zones, aiming to score by shooting into a raised net. Dribbling is not allowed.
Minigolf	Players use a putter to navigate a series of obstacles, aiming to get the ball into each hole in as few strokes as possible.
Curolling	Players slide stones across a smooth surface toward a target, aiming for precision without sweeping.

**Table 2:** Survey items and measurement scales.

Variable		Item
Media interest	1	The content of YouTube videos related to new sports is entertaining.
	2	YouTube videos related to new sports are full of positive energy.
	3	I tend to watch YouTube videos related to new sports until the end.
	4	I tend to focus when watching YouTube videos related to new sports.
Attitudes	1	Participating in new sports extremely unpleasant—extremely pleasant.
	2	Participating in new sports extremely unattractive—extremely attractive.
	3	Participating in new sports extremely worthless—extremely valuable.
Subjective norms	1	The people in my life (e.g., family/friends) would be in favor of me participating new sports.
	2	The people in my life (e.g., family/friends) would support me participating new sports.
	3	The people in my life (e.g., family/friends) would encourage me to participate new sports.
PBC	1	I have the freedom to decide when and how to practice new sports
	2	I have the ability to schedule my practice sessions according to my preferences.
	3	I feel confident in my ability to manage my new sports practice effectively.
Behavioral intentions	1	I will try to continue practicing new sports.
	2	I intend to continue practicing new sports.
	3	I am willing to devote time and effort to practicing new sports.
Social support	1	The educators show interest in my practicing new sports.
	2	The educators do not hesitate to praise me when I do well in practicing new sports.
	3	The educators are willing to help if I encounter any difficulties while practicing new sports.
	4	The educators provide guidance and feedback to help me improve my practice of new sports.

## Data Availability

Data supporting the reported results can be made available by the corresponding author upon reasonable request.
